# Vaccine hesitancy in an allergy and immunology clinic in an underserved community

**DOI:** 10.1016/j.jacig.2025.100475

**Published:** 2025-04-17

**Authors:** Albert G. Wu, William Drum, Nanette Silverberg, Khin Wathan, Han Bao, Gomeo Lam, Derek V. Chan, Mary F. Lee-Wong

**Affiliations:** a Northwell Health, Lenox Hill Hospital, New York, NY; b University of California, Santa Barbara, Goleta, Calif; c Maimonides Medical Center, Brooklyn, NY; d University of Nevada, Reno School of Medicine, Reno, Nev; e Albert Einstein School of Medicine, South Bronx, NY; f Philadelphia College of Osteopathic Medicine, Georgia Campus, Suwanee, Ga; g All Dermis Dermatology, New York, NY; h Icahn School of Medicine at Mount Sinai, New York, NY

**Keywords:** Vaccine hesitancy, COVID-19, influenza, public health, pandemic, immunization, outpatient, health care barriers, minority

## Abstract

**Background:**

Allergy and immunology patients may be immunocompromised and at higher risk for infections. Identifying vaccine-hesitant groups is essential to protecting these individuals and those at risk in the community.

**Objective:**

We analyzed patient characteristics and factors associated with influenza and coronavirus disease 2019 (COVID-19) vaccine hesitancy in a clinic in a diverse community.

**Methods:**

Vaccination history and demographic information were collected as part of intake questionnaires from 338 new patients presenting to an outpatient allergy/immunology clinic from March 2023 to February 2024. Data were analyzed by Microsoft Excel and RStudio.

**Results:**

There were 94 men and 244 women (median [range] age, 41 [18-93] years). Ethnicities included 126 White (37.2%), 107 Hispanic (31.6%), 64 Asian (18.9%), and 41 Black (12.1%). English primary language speakers comprised 80% of the patient group. In this study, 39 subjects (11.54%) refused both influenza and COVID-19 vaccines; of these 39, the distribution in each category was as follows: 22 White (17.5%), 12 Hispanic (11.4%), 3 Asian (4.76%), and 2 Black (4.9%). Sex, age, and language were not significantly associated with vaccine refusal rate. Ethnicity was found to be significant. In our study, self-identified non-White patients were less likely to demonstrate vaccine hesitancy than the self-identified White patient group (odds ratio, 0.41; 95% confidence interval, 0.21-0.81).

**Conclusion:**

Many publications report minorities are more prone to vaccine hesitancy, hindering herd immunity. Odds ratios for refusal of both vaccines were lower for minorities in this studied population. Focusing efforts on all patients may enhance vaccination initiatives.

Vaccine hesitancy is defined by the World Health Organization as a delay in acceptance or refusal of vaccines despite the availability of vaccine services and supporting evidence.^1^ Vaccine hesitancy is an active, annual discussion in health care. In recent years, the influenza and coronavirus disease 2019 (COVID-19) vaccinations have dominated discussions. Influenza, collectively referring to the multitude of infectious strains of viruses, is a droplet- and airborne-spread pathogen responsible for over 31 million illnesses, 350,000 hospitalizations, and 20,000 deaths annually in the United States,^2^^,^^3^ as well as a source of considerable burden to emergency services. Annual vaccinations are designed to cover the predominant strains of the virus for the flu season. In the northern hemisphere, virus activity peaks between December and February, although activity can begin as early as October and last as late as May.^4^

COVID-19, caused by variants of severe acute respiratory syndrome coronavirus 2 virus, was responsible for over 1 million deaths in the United States alone since 2019.^5^ Vaccines developed for this virus utilize several antigens to elicit immunization responses, including mRNA, spike proteins, and viral vectors.^6^^,^^7^ Many public resources are committed to encouraging immunization for both flu and COVID-19. However, from 2020 to 2022, the United States saw a decline in the vaccination rate of the influenza vaccine.^8^ Vaccine efficacy aims to achieve herd immunity,^9^ so it is in the public health interest to increase rates of immunizations. We elected to do a quality improvement project directed toward the improvement of vaccination rates in an outpatient clinic setting using a two-phase design. The first phase identified characteristics of patients more prone to vaccine hesitancy. The second phase applied successful initiatives learned from the COVID-19 pandemic to increase immunization rates in this vulnerable population.

## Methods

We implemented a quality improvement project at an outpatient allergy and immunology center in Brooklyn, New York. The clinic and its affiliated medical center serve a diverse population with among the lowest vaccination rates of the 5 boroughs^10^ and includes the least vaccinated zip code in New York City.^11^ The goal of the project was to improve the vaccination rate for influenza and COVID-19 for clinic patients. The first phase screened patients who demonstrated vaccine hesitancy to identify shared characteristics. During their visit, standardized questions were used to query patients about their vaccination status for the influenza vaccine and the COVID-19 vaccine for the 2023 year, as well as if they had been previously vaccinated for influenza. Query data and patient demographics were compiled in Excel and analyzed by RStudio. The study was exempted by the institutional review board at Maimonides Medical Center.

## Results

The clinic saw 338 patients over 1 year (March 2023 to February 2024). The patients included 94 male (27%) and 244 female (73%) patients with a mean age of 44 years and a median of 41.5 years. White individuals made up the plurality of patients (43%, 126), followed by Asian (15%, 46) and Black (14%, 41) individuals (Table I). While most patients (81%, 276) spoke English, 19% of patients listed a different primary language. Eleven non-English primary languages were represented, including Spanish, Chinese, Bengali, and Yiddish.

**Table I:** Patient demographic data by proportion of influenza and COVID-19 vaccination hesitancy

Characteristic	No. (%)	Influenza vaccine hesitant	COVID-19 vaccine hesitant
Total population	338	168 (49.7)	51 (15.1)
Sex			
Male	94 (35.1)	48 (51.1)	15 (16.0)
Female	244 (64.9)	120 (49.2)	36 (14.8)
Age			
0-20 years	14 (4.1)	7 (50.0)	3 (21.4)
21-40 years	146 (43.2)	82 (56.2)	23 (15.8)
41-60 years	115 (34.0)	53 (46.1)	14 (12.2)
61+ years	63 (18.6)	26 (41.3)	11 (17.5)
Primary language			
English	276 (81.7)	142 (51.4)	43 (15.6)
Not English	62 (18.3)	26 (41.9)	8 (12.9)
Spanish	22 (6.5)	8 (36.4)	1 (4.5)
Chinese	13 (3.8)	5 (38.5)	1 (7.7)
Other	28 (8.3)	13 (46.4)	6 (21.4)
Ethnicity			
White	126 (43.4)	70 (55.6)	29 (23.0)
Asian	46 (15.9)	14 (30.4)	1 (2.2)
Black	41 (14.1)	22 (53.7)	4 (9.8)
Other	125 (43.1)	62 (49.6)	17 (13.6)

Data are presented as nos. (%).

**Fig 1:**
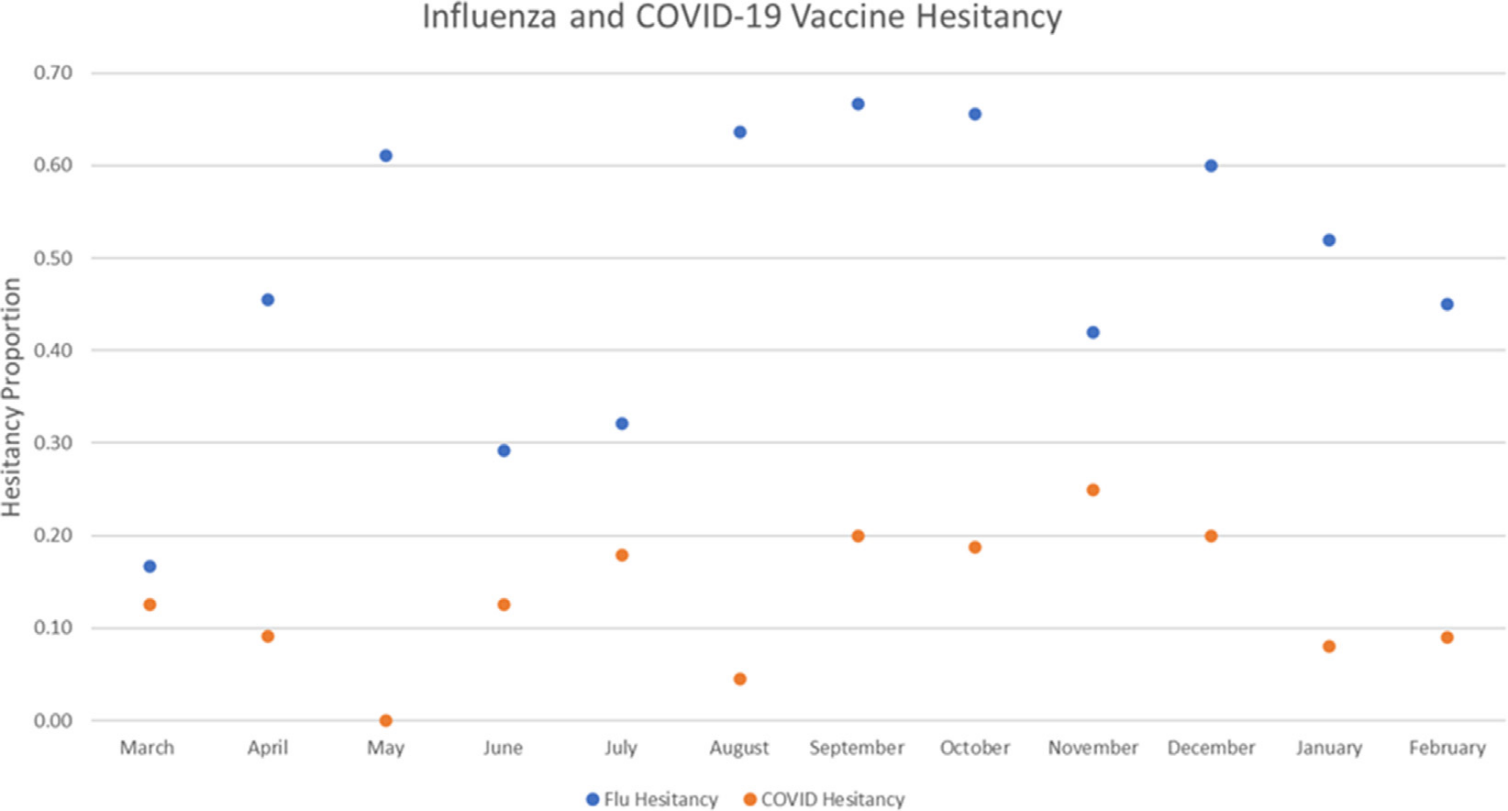
Monthly rates of influenza and COVID-19 vaccination hesitancy over 1 year. While rates of COVID-19 vaccination hesitancy remained relatively consistent throughout, influenza hesitancy showed more variability and bimodal distribution, with peaks in March and July.

Overall, 53.6% of patients demonstrated some vaccine hesitancy; 49.7% of patients seen at the clinic demonstrated vaccine hesitancy for the influenza vaccine, 15.0% for the COVID-19 vaccine, and 11.5% for both. While rates of COVID-19 vaccination hesitancy remained relatively consistent throughout the year ($\bar{x}$ = 15%, range 0-24%), influenza hesitancy showed more variability ($\bar{x}$ = 48%, range 17-67%) and a bimodal distribution, with peaks in March and July (Fig 1).

Hesitancy rates were 40-50% for all age groups except patients above 60, who demonstrated a relatively lower flu vaccination hesitancy rate of 29.7%. Prior vaccination was also associated with decreased hesitancy, as 87.0% of patients who accepted the influenza vaccine this year had received it the previous year. Correspondingly, 75.9% of patients who declined influenza vaccination this year had not received it last year. Primary English–speaking patients were overall more vaccination hesitant than non–primary English–speaking patients (51.4% to 41.9%, respectively). Demographically, patients identifying as Asian (including Chinese, Filipino, Indonesian, and Bangladeshi) demonstrated below-average hesitancy for the influenza vaccine (30%). We found no significant difference between sexes for vaccination rates or hesitancy.

## Discussion

Vaccine hesitancy is not a novel phenomenon but has experienced increased media attention and popular culture discussion over the last few years, especially in the wake of the COVID-19 pandemic. It is complex, and multiple studies have attempted to elucidate the individual, population, temporal, and contextual factors that contribute to its manifestation. Mistrust of authority is among the most cited reasons by patients for vaccination hesitancy,^12^ including decreased confidence in the health care system and the government, as well as reservations regarding the safety and efficacy of vaccinations. Studies have implied variance in borough-based hesitations. The individuality of neighborhoods—including perceived threats, behavioral control, and autonomy—are all known to affect vaccination tendencies. With this implication, health care workers are also varied in their vaccination status, which inevitably skews patient confidence. Although medical practitioners including physicians, physician associates, and nurses were among the highest in vaccinated staff, many addressed COVID-19 directly by demonstrating the highest hesitancy. This implicates another formulation of theory where core beliefs may not necessarily discount one’s scientific literacy, which further diversifies the approaches required for vaccination intervention.^13^

Patient ethnicity also has been identified as a factor impacting vaccine hesitancy. Demographically, non-White patients have been cited as more likely to demonstrate immunization hesitancy.^14^^,^^15^ Our data support these studies, with non-White ethnicities having above-average rates of influenza vaccine refusal except Asian patients. The nuance of preventive measures among Asian American populations is remarkably similar to Asian cultures abroad. Practices including mask wearing while ill, and past epidemic experiences have encouraged similar patterned approaches to novel phenomena such as COVID-19. In addition, an increase in xenophobia and discrimination against Asian individuals has also resulted in fear-driven prevention, including a tendency to remain at home or avoid socialization. This inevitably results in a precautionary mentality, including the means of getting vaccinated.^16^ This correlates with national trends, as among patients aged 65 and above, those identifying as White/non-Hispanic or Asian/non-Hispanic met or superseded the average vaccinated rate (55.5% and 54.5%, respectively), while patients identifying as Hispanic, Black, and other were below the average.^17^ For instance, sampling studies have implicated that many African American individuals feel distrust toward the pushing of vaccinations, as other vital medical needs in their communities had never elicited such attention. The sudden proactive approach would often come across as self-serving rather than for a collective benefit.^18^ Similarly, Haredim Jewish communities have experienced circumstances where their needs are undermined by local health officials, inevitably lessening their trust in any current or future interventions. Haredim prioritize their way of life and will work hard to maintain it, willingly facing the inevitable risks over the proposed public health benefits.^19^

Language is another barrier that contributes to vaccine hesitancy. Limited English proficiency has been associated with delays in COVID-19 immunization and increased morbidities, including hospitalizations and deaths,^20^ whereas communicating to patients in their preferred language reduces hesitancy.^21^ Interestingly, the vaccination rate of non–primary English speakers at our clinic was higher than primarily English speakers. We hypothesize this was due to the availability and implementation of interpretation services at our clinic, including the use of wheeled portable workstations, allowing for accessible interpretation in the patient’s preferred language.

Nationally, the US Centers for Disease Control and Prevention (CDC) found the overall vaccination rate for adults over 18 years to be 54% for the 2022-23 flu season.^2^ This was largely skewed by the elderly population: individuals over the age of 60 had a vaccination rate of 63.9%, while those aged 18-59 showed a rate of 35.8%.^2^ Particularly in youth, it may be presumed that vaccine hesitancy stems from the notion of having preexisting beliefs, political or otherwise, contradicted. For instance, one’s perception of whether or not a specific practice is conservative or liberal is significant enough to outweigh the preventative and cost-effective benefits of vaccination.^22^ Our study further corroborates these findings, as our most elderly patients demonstrated the lowest vaccine hesitancy rates. Medicare may play a role in this; the insurance encourages annual wellness visits as well as an initial welcome visit once patients qualify.^23^ These visits often include reviewing immunization history and age-appropriate vaccinations. Studies have shown that patients with an annual visit were more likely to receive seasonal vaccinations.^24^ An estimated 9-34% of specialists report involvement in their patients’ primary care,^25^ so these visits are opportunities for allergists to address patient hesitancy.

Finally, residing in a lower-income area is an independent factor in declining vaccination rates.^26^ This encompasses several barriers to care associated with lower income, including ease of access, limited resources, and historic undervaccination.^20^ These variables are estimated geographically by the CDC and are published as a social vulnerability index.^27^ Although we did not collect patient income data, the neighborhoods our clinic serves have higher household poverty rates than city averages.^11^

Interestingly, although the status of prior influenza vaccine appeared to contribute toward overall immunization hesitancy, the prevalence of patients who had received the COVID-19 vaccination was much higher than those who had received the influenza vaccine. We attribute this difference to the governmental and employee mandates implemented during the pandemic. While not without its drawbacks, implementing vaccine mandates is undeniably effective. Aside from the pandemic, mandates are often seen in the school-entry setting and have been instrumental in increasing childhood immunization uptake.^28^ Similarly, vaccination rates across multiple studied populations increased after COVID-19 vaccine mandates were implemented, including health care workers,^29^ public school employees, and major companies.^30^

We can take lessons from the success of the multilevel initiatives from this period and apply them to reduce influenza vaccine hesitancy.^31^ There are no mandates, but there is a concerted promotion to get immunized through advertising from the CDC, especially during peak flu season. These efforts contribute to the higher vaccination rates seen at our clinic from November to February. While effective, mass campaigns with generalized messaging are less impactful than personalized education toward patients.^32^

Our study found multiple patient characteristics differentially associated with increased vaccination hesitancy, including immunization history, age, and ethnicity. These characteristics could be used to identify patients more at risk for being vaccine hesitant going forward to make vaccination campaigns more efficient. We can also improve on current efforts to increase immunization rates by studying successful initiatives from the COVID-19 pandemic. While vaccine-hesitant patient populations may have mistrust for health care institutions, vaccinated patients show a high degree of confidence toward them.^33^ Prior initiatives aimed at improving vaccination rates have focused on educating patients^34^ and delivering consistent messaging, such as training physicians to lead individual discussions with patients.^31^^,^^35^^,^^36^

Decreasing vaccine hesitancy rests on a relationship of shared decision-making with patients.^37^ There are several methods to aid in this during routine clinic visits. For example, screening for prior vaccine hesitancy by including history items in a previsit questionnaire can risk-stratify patients. Standardized messaging and improving communication skills were demonstrated to be effective in reducing COVID-19 vaccine hesitancy rates;^38^ health care organizations should address this annually and incorporate it into training. Finally, providing educational materials for common misconceptions and concerns^34^ can improve attitudes toward vaccination acceptability.

Another potential confounder in our study includes patient comorbidities. Literature is lacking on the impact of comorbidities on vaccination rates; however, studies have looked at how the presence of specific conditions such as respiratory infections affect vaccination effectiveness.^39^ Future analyses could focus on collecting and stratifying data by income, presence of concurrent medical conditions, and education. The responses would still be subject to reporting bias, but it would permit more robust conclusions to be drawn.

In conclusion, adequate vaccination rates are vital to the public health interest to preserve the effectiveness of herd immunity, decrease disease morbidity and mortalities, and counter the spread of infections. We analyzed patients at our allergy clinic for traits associated with vaccine hesitancy. We found that patients who were noncompliant with vaccinations had a history of flu vaccine hesitancy, so a history of immunization refusal could be used as a predictor for refusal of other vaccinations. A significant number of patients who refused the flu vaccination did become vaccinated against COVID-19, suggesting that other factors, such as government or work-based mandates, could have increased uptake. As the overall vaccination rate in the United States drops, reducing misinformation about immunizations and increasing research toward vaccine hesitancy should be prioritized. Health care providers have a responsibility to be leaders in this movement for the public good. Identifying target populations for vaccine hesitancy initiatives is a vital initial step in improving long-term vaccine acceptance.

## Disclosure statement

Disclosure of potential conflict of interest: The authors declare that they have no relevant conflicts of interest.
